# Development of a Novel Strategy to Isolate Lipophilic Allergens (Oleosins) from Peanuts

**DOI:** 10.1371/journal.pone.0123419

**Published:** 2015-04-10

**Authors:** Christian Schwager, Skadi Kull, Susanne Krause, Frauke Schocker, Arnd Petersen, Wolf-Meinhard Becker, Uta Jappe

**Affiliations:** 1 Division of Clinical and Molecular Allergology, Research Center Borstel, Airway Research Center North (ARCN), Member of the German Center for Lung Research (DZL), Borstel, Germany; 2 Department of Dermatology, Allergology and Venereology, University of Luebeck, Germany; INRA, FRANCE

## Abstract

**Background:**

Peanut allergy is one of the most severe class I food allergies with increasing prevalence. Especially lipophilic allergens, such as oleosins, were found to be associated with severe symptoms, but are usually underrepresented in diagnostic extracts. Therefore, this study focused on isolation, molecular characterization and assessment of the allergenicity of peanut oleosins.

**Methods and Results:**

A comprehensive method adapted for the isolation of peanut oil bodies of high purity was developed comprising a stepwise removal of seed storage proteins from oil bodies. Further separation of the oil body constituents, including the allergens Ara h 10, Ara h 11, the presumed allergen oleosin 3 and additional oleosin variants was achieved by a single run on a preparative electrophoresis cell. Protein identification realized by N-terminal sequencing, peptide mass fingerprinting and homology search revealed the presence of oleosins, steroleosins and a caleosin. Immunoblot analysis with sera of peanut-allergic individuals illustrated the IgE-binding capacity of peanut-derived oleosins.

**Conclusion:**

Our method is a novel way to isolate all known immunologically distinct peanut oleosins simultaneously. Moreover, we were able to provide evidence for the allergenicity of oleosins and thus identified peanut oleosins as probable candidates for component-resolved allergy diagnosis.

## Introduction

The peanut (*Arachis hypogaea*) is one of the most cultivated oilseeds and a source of valuable edible oil, proteins and fiber. Peanuts contain 49% fat, 26% protein and 11% fiber [1]. However, one percent of the Western population is allergic to peanut, and the prevalence is still increasing [2]. Since diagnostic allergy testing is based on aqueous extracts of the respective allergenic source, potential allergenic proteins of lipophilic nature may be underrepresented as putative allergens [3]. Furthermore, there is a growing evidence that lipids trigger immune reactions, especially in conjunction with lipophilic proteins, pointing to the latter as potential allergens [4].

Peanut oleosins are made up of a highly conserved central hydrophobic domain and hydrophilic N- and C-termini, differing in the primary amino acid sequence. Oleosins are the major oil body (or oleosome) stabilizing proteins and assumed to be involved as enzymes in the germination process [5–7]. Oil bodies consist of a core of triacylglycerol surrounded by a single layer of phospholipids, embedded with the hydrophobic domain of the oleosins [8, 9]. Being triacylglycerol storage organelle in seeds, oleosomes can be found among a variety of oil-rich plants [8, 10, 11]. In contrast to peanut, the allergenic character of oleosins derived from sesame seeds and hazelnut and their association with serious symptoms has been clearly shown [3, 12, 13]. At least eight peanut-derived oleosins have been identified on the DNA level (www.uniprot.org) and proteomic approaches so far [14, 15]. Resulting from different molecular masses, due to the insertion of additional amino acid residues in the C-terminal domain, a classification into high- or low-M_r_ isoforms has been established [16, 17].

Two peanut (*Ara* *chis* *h* *ypogaea*) oleosins, Ara h 10, as well as an isoform, and Ara h 11 were approved by the WHO/IUIS-Allergen Nomenclature Subcommittee (www.allergen.org) as allergens in 2008, but the respective data have not yet been published (February 2015). Concerning the oleosin variant A (Uniprot accession no. Q9AXI1) Pons et al. [18] were able to show IgE-reactivity, but this finding has not been confirmed by others. Beside another isoform (oleosin variant B), which shows a sequence identity of 96% to variant A, an additional isoform, termed oleosin 5, was discovered by our group in 2004 (Uniprot accession no. Q6J1J8). Recently, Kobayashi et al. were able to identify an IgE-reactive epitope on a peptide of oleosin 3 (Uniprot accession no. Q647G3), obtained after enzymatic hydrolysis of peanut total protein, showing cross-reactivity with buckwheat [19].

Interestingly, Olszewski et al. were able to identify peanut-allergic patients reacting to oleosins present in refined peanut oil [20]. Peanut oil has been used as an essential ingredient in different skin care products like face creams, massage oils or ointments for the treatment of atopic eczema [21]. In the light of an ongoing controversial discussion concerning the skin as a possible sensitization route, it is important to know whether refined peanut-derived products (e.g. oil, lecithin) contain lipophilic allergens including oleosins [20–23]. Furthermore, the biotechnological advances of natural and reconstituted oleosomes have been explored regarding their chemical properties for a possible utilization in industry over the last years [6, 10, 24, 25]. Serving as natural emulsifiers or as carriers for active agents, different applications such as foodstuffs, personal care products and pharmaceuticals could be addressed in the future [26–28].

As the allergenicity of peanut oleosins is still a matter of debate, a comprehensive investigation of the molecular characteristics, the immunological features and the isolation conditions is consequently necessary to determine the allergenic potential of these proteins. The biggest problem to overcome is the low solubility of oleosins in aqueous solutions and organic solvents. Hence, the use of strong detergents is needed, which restricts the number of separation techniques considerably. In order to generate highly-sensitive analytical tools to detect oleosins in all kinds of matrices, these problems must first be solved.

In this study, we were able to establish a new method for the simultaneous isolation of all known immunologically distinct peanut oleosins.

## Materials and Methods

### Preparation of the peanut extract

Roasted peanuts (‘Seeberger Riesen’, Seeberger, Ulm, Germany) were bought in a local supermarket. Unroasted peanuts were purchased in a pet store. Peeled kernels were placed in a mortar, covered with liquid nitrogen and afterwards homogenized using a grinder (Moulinex, model AR100, Alencon, France) for 40 s. Ground peanuts (50 g) were suspended in 200 mL of 0.1 M potassium phosphate buffer, pH 7.2, containing 0.33 M sucrose and stirred for 30 min at room temperature.

### Isolation of peanut oil bodies

After filtration, isolation of oil bodies was conducted according to the protocol of Millichip et al. [29] with the following modifications. The homogenate was placed in a 250 mL centrifugation bottle (Thermo Fisher Scientific, Waltham, MA, US) and centrifuged at 16,000 g (15 min, 20°C) (Herolab, model HiCen 21C, Wiesloch, Germany). The oil bodies, which formed a white fat pad floating on the supernatant, were dissolved in 5 mL of a freshly prepared 50 mM Tris/HCl buffer, pH 7.2, containing 9 M urea, by stirring for 10 min. The resuspension was again centrifuged (10,000 g, 15 min, 20°C) in a 50 mL centrifugation tube (Roth, Karlsruhe, Germany) and the fat pad was recovered. This step was repeated three more times. The obtained fat pad was further purified using detergent washing, ionic elution and integrity testing by hexane [30]. Briefly, the collected fat pad was dissolved in 20 mL detergent washing solution (0.1% Tween 20, 0.2 M sucrose, 5 mM sodium phosphate buffer, pH 7.5) and placed at the bottom of a 50 mL centrifugation tube. Afterwards, 10 mL phosphate buffer (10 mM, pH 7.5) was layered on top, and the suspension was centrifuged (10,000 g, 15 min, 20°C). Again, the floating oil bodies were recovered and suspended in 20 mL ionic elution buffer (2 M NaCl, 0.6 M sucrose, 10 mM phosphate buffer, pH 7.5), and overlaid with ionic elution buffer containing 0.25 M instead of 0.6 M sucrose. The centrifuged oleosomes were collected, mixed with 20 mL phosphate buffer (10 mM, pH 7.5) and 20 mL hexane. After centrifugation, the hexane layer was discarded and the oil bodies recovered once again.

### Delipidation of the oil bodies

The isolated oleosomes were placed in a 50 mL centrifugation tube and mixed with 3-fold excess of cold acetone to break up the oil body and release neutral lipids. After centrifugation (10,000 g, 5 min, 4°C), precipitated proteins were recovered and washed with acetone another three times. Proteins were then dried under nitrogen and stored at −80°C until use.

### Purification of peanut oil body proteins

Oleosins were purified from the precipitated proteins using the Bio-Rad Model 491 Prep Cell (Bio-Rad, Hercules, CA, US) with continuous elution system. Electrophoresis was carried out applying the buffer conditions of the modified tricine-SDS-PAGE protocol of Haider et al. [31]. A 10% (w/v) polyacrylamide resolving gel (7.0 x 3.7 cm ID; 19:1, acrylamide-bis, Roth, Karlsruhe, Germany) was topped with a 4% stacking gel. Extracted oil body proteins (10 mg) were solubilized in 300 μL of a 0.1 M phosphate buffer containing 3% SDS. Afterwards, 500 μL 5x reducing SDS-PAGE sample buffer and 200 μL deionized water were added. The solution was mixed vigorously, heated for 5 min at 95°C and applied onto the gel column. Electrophoresis was performed with a constant current of 50 mA (180–350 V). After elution of the ionic front, fractions of 2 mL were collected at a flow rate of 0.5 mL/min using a Fast Protein Liquid Chromatography system (Äkta prime, Amersham Bioscience, Fairfield, CT, US). Protein elution was monitored at 280 nm, every 10th fractions was analyzed by SDS-PAGE and stained with the sensitive colloidal Coomassie staining protocol of Kang et al. [32] to determine the fractions containing the desired proteins. For the concentration of proteins (5:1), 1 mL of each fraction was transferred into an ultra-centrifugal device with a 3 kDa molecular weight cutoff (Merck, Darmstadt, Germany). Protein concentration was determined using the Bradford assay with BSA as standard (Thermo Fisher Scientific, Waltham, MA, US).

### SDS-PAGE and Western blotting

SDS-PAGE was conducted according to Laemmli et al. [33] using the XCELL Mini Cell System (Novex, San Diego, CA, US) with 12% polyacrylamide gels (Life Technologies, Carlsbad, CA, US) under reducing conditions. After electrophoresis, proteins were visualized by staining with colloidal Coomassie. Immunoblotting was carried out using the semi-dry blotting technique [34]. Proteins were transferred at 0.8 mA/cm^2^ for 45 min onto a methanol-activated polyvinylidene fluoride (PVDF) membrane (pore size 0.45 μm, Millipore Corporation, Billerica, MA, US), which was blocked thereafter with TTBS (Tris buffered saline with Tween 20; 100 mM Tris, 100 mM NaCl, 2.5 mM MgCl_2_, 0.05% Tween 20, pH 7.4) supplemented with 2.5% skimmed milk powder. Membrane strips were incubated with diluted patients’ sera (1:10 in TTBS, with 2.5% skimmed milk) or anti-oleosin antibodies [35] (1:5,000 in TTBS, with 2.5% skimmed milk) overnight. Detection of bound antibodies was conducted using alkaline phosphatase-conjugated secondary antibodies, mouse anti-human IgE (Pharmingen, San Diego, CA, US, 1:10,000) or goat anti-rabbit IgG (Jackson Immuno Research, West Grove, PA, US, 1:5,000), diluted in TTBS for 2 h. Immunostaining was carried out according to Blake et al. [36]. Blotted proteins were stained with 0.1% Coomassie in 50% methanol and Gold solution (Sigma Aldrich, St. Louis, MO, US).

### Protein sequencing

After electrophoresis, N-terminal microsequencing was performed as described elsewhere [37]. Protein transfer was achieved by semi-dry blotting onto PVDF membrane using 10 mM *N*-cyclohexyl-3-aminopropanesulfonic acid with 10% methanol (pH 11.0) as transfer buffer. Afterwards, the membrane was washed with deionized water and stained by 0.1% Coomassie in 50% methanol, destained in 50% methanol and air dried. Excised protein bands were sequenced using a Procise protein sequencer with online PTH (Phenylthiohydantoin) amino acid analyzer (PE Biosystems, Weiterstadt, Germany).

### In gel digestion of proteins and peptide mass fingerprinting

Coomassie-stained protein bands were excised and prepared as described [38]. Briefly, excised and chopped bands were washed, destained and digested by trypsin (Trypsin Gold, Mass Spectrometry Grade, Promega, Mannheim, Germany) overnight. The ZipTip (C_18_, Millipore Corporation, Billerica, MA, US) eluates of the obtained tryptic fragments were mixed 1:1 (v/v) with a 12 mg/mL α-cyano-4-hydroxycinnamic acid matrix (Bruker Daltonics, Bremen, Germany), dissolved in a 2:1 (v/v) mixture of 100% acetonitrile/0.3% TFA, and spotted on the target. Tryptic mass fingerprinting was performed as described previously [39] using a Reflex III (Bruker Daltonics, Bremen, Germany) in reflector mode, while applying an acceleration voltage of 20 kV. External mass calibration was performed with peptide standard II (Bruker Daltonics, Bremen, Germany). Mascot Peptide Mass Fingerprint (http://matrixscience.com) and NCBInr database were used to identify digested fragments. For database search the following filters were applied: taxonomy on other green plants, peptide tolerance of ± 0.3 Da and up to one allowed missed cleavage. Variable modifications were the oxidation of methionine residues and N-terminal acetylation.

### Human sera and rabbit antiserum

Sera of four patients with a positive clinical history of peanut allergy were used for the evaluation of IgE reactivity of purified peanut oleosins in immunoblot analysis. Sera of a healthy individual and an allergic patient (inhalant allergy) without symptoms after consumption of peanuts and without sensitization to peanut were used as controls. All patients who were known to be allergic to peanut refused to have oral challenge tests with foods, due to severe reactions in their history.

The anti-oleosin antibody was obtained after the immunization of rabbits with a multi-antigenic peptide (MAP) with the following sequences: VQVHTPTTQRVDVPR and MADYVGQKTKDAGQQ taken from Q9AXI1 [35]. These sequences are located in the N- and C-terminal domain of oleosin 5 and oleosin variant A. Similarly, these sequences can be found in oleosin variant B, but with one replaced amino acid in each sequence. Additionally, MADYVGQKTKDAGQQ shows an identity of 76% to a sequence in both Ara h 10 isoforms and enables their detection.

### Ethics Statement

The research was conducted according to the principles expressed in the Declaration of Helsinki, and approved by local ethics committee of the University of Luebeck (approval number 10–126). All patients gave a written informed consent.

The immunization of the animals was carried out in strict accordance with the recommendations in the Guide for Care and Use of Laboratory Animals of the National Institutes of Health. Due to German animal protection law, the permission for animal experiments was given by the governmental animal welfare committee which in our district belongs to the Landesamt für Natur, Umwelt und Verbraucherschutz, Nordrhein-Westfalen (Seibertzstraße 1, 59821 Arnsberg, Nordrhein-Westfalen) (reference number: 23.8720 Nr. S-anzeige 11) which is the successor of the Bezirksregierung Arnsberg. In order to achieve enough blood it was necessary to sacrifice the rabbits by exsanguination after anesthesia. The anesthesia was performed with Ketaminhydrochlorid (Ketanest, Parke-Davis, Berlin, Germany) and Xylazin (Rompun, Bayer, Leverkusen, Germany). Before performing the injection of the antigen-adjuvant emulsion in 4 volumes of approximately 200 μl per injection near the dorsal shoulders, the site was locally anesthetized with Lidocainhydrochlorid (Xylocain, Astra Zeneca, Södertälje, Sweden). Local anesthesia with Lidocainhydrochlorid of the ear was also performed before blood sampling from the rabbit (500 μl from the ear vein).

## Results and Discussion

### Isolation of oil body proteins

Oleosins are the most abundant oil body proteins, comprising of a hydrophobic domain which is embedded into the oil body matrix, and an amphipathic N- and C-terminal domain covering the entire surface of the oleosome [14]. It has been suggested that at neutral pH the positively charged amino acids of the amphipathic domains are directed towards the negatively charged head groups of the phospholipids, whereas the negatively charged amino acids are directed towards the exterior [40, 41]. Thus, the steric hindrance and the negative charge prevent the coalescence of oil bodies [17, 42]. However, the electrostatic repulsion depends on both, the pH and the ionic strength of the aqueous environment [43, 44]. At a pH close to the isoelectric point of the oleosomes (between pH 5–6) aggregation starts due to an attenuation of the electrostatic repulsion [43–45]. The same effect can be observed with increasing salt concentrations as cations (e.g. Na^+^, Ca^2+^) are able to shield the electrostatic repulsion of the negatively charged amino acids on the oil body surface [44, 46]. However, this oleosome aggregation seems to be reversible [42].

**Fig 1:**
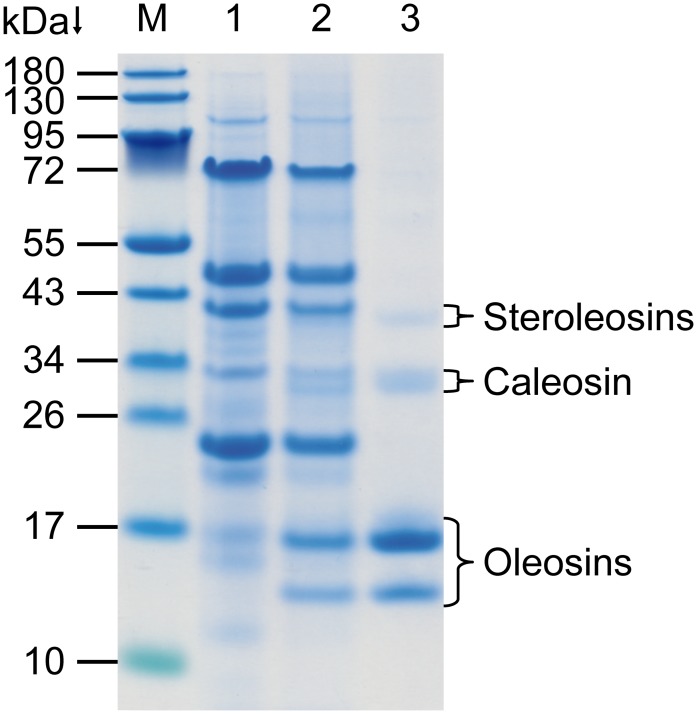
Purification steps of the oil body isolation from unroasted peanuts shown by SDS-PAGE. M, molecular mass marker; lane 1, total extract; lane 2, fat pad obtained after centrifugation of the total extract; lane 3, purified oil bodies.

Isolation and purification of oil bodies from unroasted peanut seeds were conducted for structural characterization by centrifugation-flotation, treatment with urea, detergent washing, ionic elution and integrity testing by use of hexane (Fig 1). In particular, the neutral pH of the solutions used provided a fully accessible surface of the individual oil bodies and supports the separation of non-specifically associated proteins in this step by step procedure. Additional washing with cold acetone facilitated the isolation of oil body proteins by way of the oil body disintegration and lipid removal. The fat pad obtained after the first centrifugation of the total extract (Fig 1, lane 2) differs from the protein composition of the initial extract (Fig 1, lane 1) especially between 14–17 kDa. Owing to the specific molecular masses these proteins are assumed to be oleosins [14, 15]. Further washing removed most of the seed storage proteins (globulins and albumins) and intensified these oleosin bands along with three pale protein bands of about 30 kDa and 40 kDa, confirming that these proteins are constituents of the oleosomes (Fig 1, lane 3). This finding is in accordance with the recent discovery of minor oil body proteins called caleosins and steroleosins in diverse species, comprising of a molecular mass of about 30 kDa and 40 kDa, respectively [47–49].

### Purification of oil body proteins

An additional problem beyond the close molecular masses which must be overcome while purifying oleosins is their tendency to interact with each other due to their hydrophobic nature. The interaction leads to the formation of self or mixed aggregates, even when applying strong denaturing conditions, and prevents a separation with powerful HPLC procedures, leading to co-elution and elution of micellar aggregates [29, 50]. High ionic strength even facilitates the dimerization and oligomerization reactions [50, 51]. In general, the use of strong detergents to prevent aggregation and to keep oleosins in solution is crucial. Experiments performed by Kim et al. demonstrated that a phosphate buffer (well known for its solubilization properties), supplemented with SB 3–10 (zwitterionic detergent), NP-40 or dodecyl-maltoside (both non-ionic detergents) had the most significant effects on the solubility of oleosins, whereas other kinds of detergents (e.g. Brij 56, CHAPS) were less effective [52, 53]. In some cases the addition of chaotropes like urea and thiourea or 10% glycerol further enhanced the solubility of oleosins [52, 53]. Similarly, the anionic detergent SDS is able to solubilize crude oil body proteins and to impede the dimerization processes, even when the solution is stored several months (own observations). Since the application of detergents on HPLC systems causes often problems, purification of the oil body proteins was conducted using a continuous-elution electrophoresis cell combined with the low salt concentration protocol developed by Haider et al. [31].

**Fig 2:**
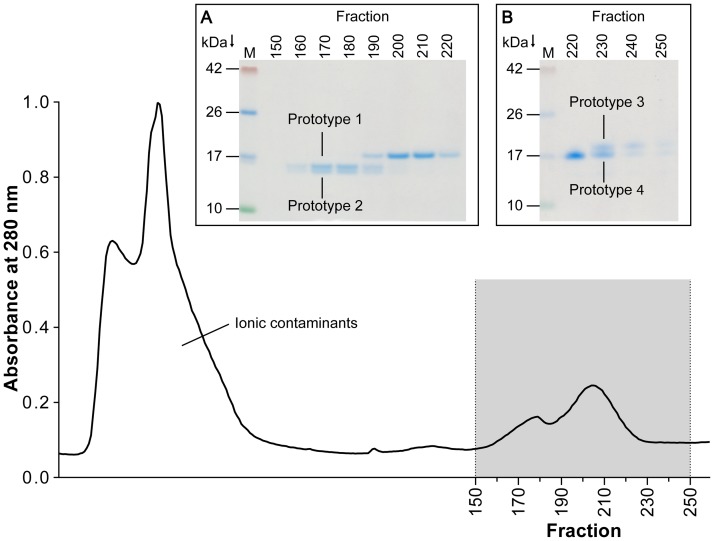
Representative elution profile of oil body proteins separated by preparative electrophoresis monitored at 280 nm. Electrophoresis was performed with extracted oil body proteins on a 10% polyacrylamide resolving gel, and fractions of 2 mL were collected. Fractions corresponding to the main peaks were visualized by SDS-PAGE on 12% polyacrylamide gels with Coomassie staining (Insert). M, marker; **(A)** Fraction 150–220 **(B)** Enriched (5:1) fractions 220–250, for prototype classification see Table 1.

After elution of the ionic front and dye, a peak occurring at fraction 160 indicated the elution of two oleosins with molecular masses of roughly 15.5 kDa and 16 kDa as shown by SDS-PAGE analysis (Fig 2, insert A). A progressive transition between these proteins and an additional protein of approximately 17 kDa was monitored at fraction 190, whereas successive fractions only contained the protein of higher molecular weight. A further transition of proteins was observable only after concentrating fractions 220-250 (5:1) (Fig 2, insert B), showing a protein comprising of a molecular mass of about 17.5 kDa. In contrast to the findings of Pons et al. [49], our results show that the oleosin variants A and B (17.5 kDa band) are not the major peanut oleosins and indicate an almost equal distribution of all oleosins among the oleosome. Previously published data also support our results [15].

Isolation of single compounds by using altered conditions was not successful (data not shown) owing to the limited resolution of the Prep Cell. Nevertheless, the separation achieved was sufficient to identify the different oleosins in the fractions by peptide mass fingerprinting, N-terminal sequencing and specific antibodies. Thus, the requisite to investigate the specificity of patient-IgE is given.

A classification of peanut oleosins into high and low molecular isoforms according to Tzen [16] has been arranged by Jolivet et al. [14]. Although this clustering might be appropriate for a basic molecular and immunological differentiation of oleosins [54], it does not reflect the immunogenic properties of a group of eight peanut-derived oleosins. Therefore, we propose a new classification model (Table 1) based on the sequence identity, contributing to the fact that similar sequences are recognized equally by patients’ IgE. This model clusters the peanut oleosins in four distinct groups of immunogenic related compounds called ‘prototypes’.

**Table 1:** Classification of peanut oleosins.

Name	Uniprot accession no.	Isoform [14]	Number of amino acids
**Prototype 1**			
Ara h 10.0101	Q647G5	H-oleosin	169
Ara h 10.0102	Q647G4	H-oleosin	150
**Prototype 2**			
Ara h 11.0101	Q45W87	L-oleosin	137
Isoform of Ara h 11.0101	Q45W86	L-oleosin	137
**Prototype 3**			
Oleosin variant A	Q9AXI1	H-oleosin	176
Oleosin variant B	Q9AXI0	H-oleosin	176
Oleosin 5	Q6J1J8	H-oleosin^1^	176
**Prototype 4**			
Oleosin 3	Q647G3	L-oleosin	166

^1^ not classified by Jolivet et al. [14], but in analogy to the oleosin variants A and B

### Characterization of the oleosins by peptide mass fingerprinting and N-terminal sequencing

**Fig 3:**
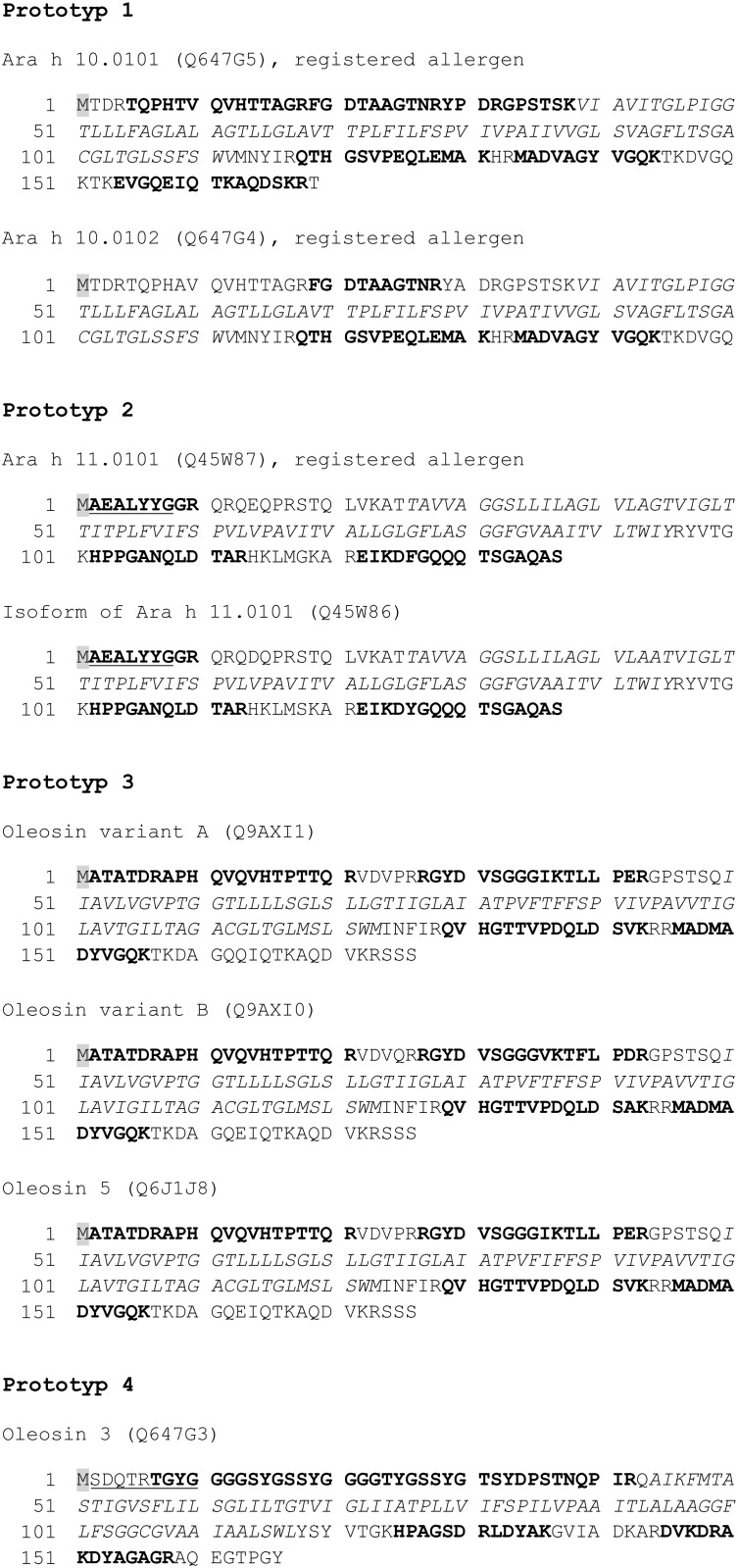
Identification of peanut oleosins using peptide mass fingerprinting and N-terminal sequencing. Peptides resulting from tryptic digestion were searched against the NCBInr database using the Mascot search engine. Bold letters indicate identified sequences by MALDI-TOF-MS, whereas underlined letters mark sequences obtained after N-terminal sequencing. The hydrophobic domains, not accessible to tryptic cleavage, are written in italics. The methionine residue, highlighted in grey, was not observed.

Using tryptic mass fingerprint analysis and N-terminal sequencing including homology search, we were able to identify oleosins from each prototype (Fig 3). Due to the cleavage by trypsin exclusively in the C-terminal region of arginine and lysine residues, the hydrophobic central domain is inaccessible for enzymatic digestion due to a lack of cleavage sites [55]. Thus, cleavage is only possible in the hydrophilic C- and N-terminal region of the oleosins, reducing the overall coverage of the protein to approximately 50%. However, a certain number of peptide fragments resulting from specific cleavage has been identified for each oleosin of the different prototypes. In addition to that, acetylation of the N-terminal domain of oleosins was observed by MALDI-TOF-MS (mass difference: 42 Da), except for prototype 1 and prototype 4. In contrast to Lin et al. [56], oleosin fragments without acetylation were also observable among the different prototypes, but to a much lesser extent. Thus, N-terminal sequencing of the prototypes 2 and 4 was successful, revealing the sequences AEALYYG and SDQTRTGYG, respectively (Fig 3). However, the N-terminal methionine residue was neither detected with MALDI-TOF-MS nor N-terminal sequencing, which corresponds to previously published data of sesame oleosins [56]. A translational modification of the N-terminal domain of Ara h 10 has been described [14], but the peptide fragment carrying a possible acetylation was not detected in our experiments. Acetylation of the N-terminal domain is a common posttranslational modification in eukaryotic organisms [57, 58], regulating enzymatic activities [59], increasing thermal stability [60] and preventing ubiquitin-dependent degradation [60, 61]. Since oleosomes are designated for long-term storage of lipids, the preservation of oleosins is a key element to maintain the integrity of oil bodies.

Moreover, we were able to prove the existence of a peanut caleosin by a proteomic approach for the first time (S1 Fig) and verified the presence of two peanut steroleosins (Uniprot accession no. A7LB59, A7LB60) (S2 Fig).

### Immunological recognition of peanut oleosins

**Fig 4:**
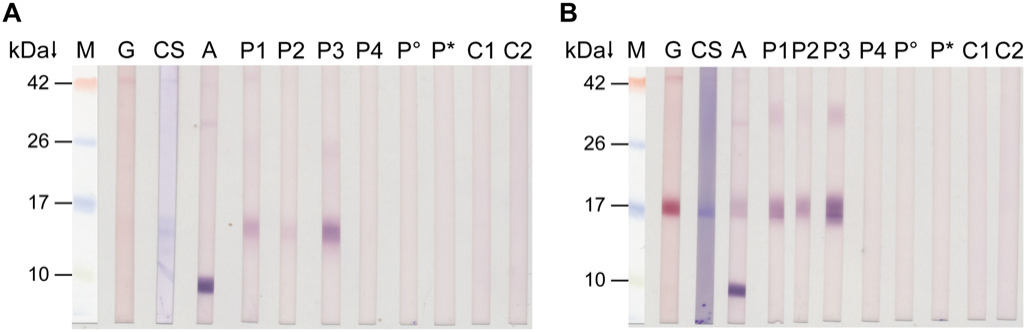
Western blot of oleosin prototypes isolated from roasted peanuts and purified by preparative electrophoresis. Fractions obtained by preparative electrophoresis containing the prototypes 1/2 (A) and prototypes 3/4 (B) were pooled, subjected to SDS-PAGE and subsequent immunoblotting. M, molecular mass marker; G, Gold staining; CS, Coomassie staining; A, polyclonal anti-oleosin antibody; P1–P4, allergic patients’ sera; P°, allergic individual (not to peanut) with respiratory symptoms; P*, healthy control; C1, secondary antibody control (mouse anti-human IgE antibody); C2, secondary antibody control (goat anti-rabbit IgG antibody).

For the assessment of the allergenic properties of the oleosin prototypes, roasted peanuts were chosen, following the developed isolation strategy, because in the western countries peanuts are usually consumed after roasting. Pooled fractions of prototype 1/prototype 2 and prototype 3/prototype 4 were subjected to SDS-PAGE and subsequent Western blotting (10 μg/cm). Sera from four peanut-allergic individuals were screened for IgE reactivity (Fig 4). Interestingly, all four prototypes were recognized exclusively by the sera of three patients (Fig 4A and 4B, lane P1–P3) with a clinical history of severe anaphylactic reactions to peanut. The symptoms included anaphylactic shock, cardiac problems, generalized urticarial, dyspnea, hypotonia, angioedema, laryngeal edema, flush, with minor differences between the three patients. The fourth peanut-allergic patient with moderate to severe symptoms did not display a reaction to any oleosin prototype at all (Fig 4A and 4B, lane P4). This can be explained by the fact that in this case the peanut allergy is pollen-associated, which is due to the cross-reactivity between birch pollen and peanut, mainly via the Bet v 1-homologue Ara h 8.

Apart from that, detection of proteins of approximately 10 kDa (Fig 4A and 4B, lane A) and 34 kDa has also been observed (Fig 4B, lane A, P1, P3). This shows on the one hand the tendency to decompose into smaller fragments as examined previously [49] and on the other hand the potential to form dimers and oligomers as reported for oleosins from diverse species [49, 51, 62]. Using autoradiography Pons et al. [49] detected a 6 kDa fragment, resulting from the cleavage at the C- or N-termini, but they were not able to observe the corresponding counterpart. Since our anti-oleosin antibody recognized a protein band at 10 kDa, we suggest that this fragment could be the corresponding protein fragment (Fig 4A and 4B, lane A). However, it does not seem to possess immunogenic features for the peanut-allergic patients, as no reaction has been detected so far. The weak reaction to an additional higher molecular mass band at about 34 kDa (Fig 4B, P1 and P3) is likely due to the formation of self or mixed dimers, even occurring when oleosins were stored in detergent-containing solutions.

Although only a small number of peanut-allergic individuals could be tested, we were able to show that, in addition to the accepted allergens (prototype 1 and 2), other oleosins (prototype 3 and 4) are potential allergens possibly associated with severe reactions.

## Conclusion

To summarize the major results of this study, a method has been developed that allows the separation of all peanut oleosins described so far. In addition, further oil body constituents, a caleosin and two steroleosins, were isolated and identified. Immunoblot analyses revealed the binding of IgE antibodies in sera of peanut-allergic patients to all prototypes, providing evidence for the allergenicity of the corresponding oleosins. More patients with different clinical phenotypes need to be investigated to expand the knowledge on oleosin allergenicity.

The future component-resolved diagnostics will be performed with recombinant proteins due to obvious advantages of this production technique. The purified natural oleosins provide an excellent tool for the authentication of the recombinant surrogates.

Concerning applications in the food, cosmetic or pharmaceutical industry, a hypoallergenic oleosin respective oleosome would provide an enormous benefit. However, since the testing of the allergenicity of oleosins has just begun, there is a lack of data with respect to the safe use of oleosins for industrial purposes. Aside from that, genetic engineering is used to change the properties of both, the hydrophobic domain and the amphipathic domains by varying amino acid sequences or truncating the central hydrophobic domain to produce specialized surfactants [63]. The overall effect on the allergenicity of these modified oleosins is not predictable and needs to be further investigated in order to establish a safe tool for the industrial use.

## Supporting Information

S1 Fig Identification of a peanut caleosin using peptide mass fingerprinting. (TIF)

S2 Fig Identification of peanut steroleosins using peptide mass fingerprinting and N-terminal sequencing. (TIF)
