# Supplementary figures and images for: Development of a Novel Strategy to Isolate Lipophilic Allergens (Oleosins) from Peanuts
Source: PLoS One. 2015 Apr 10;10(4):e0123419. doi: 10.1371/journal.pone.0123419 (PMC4393030; doi:10.1371/journal.pone.0123419)

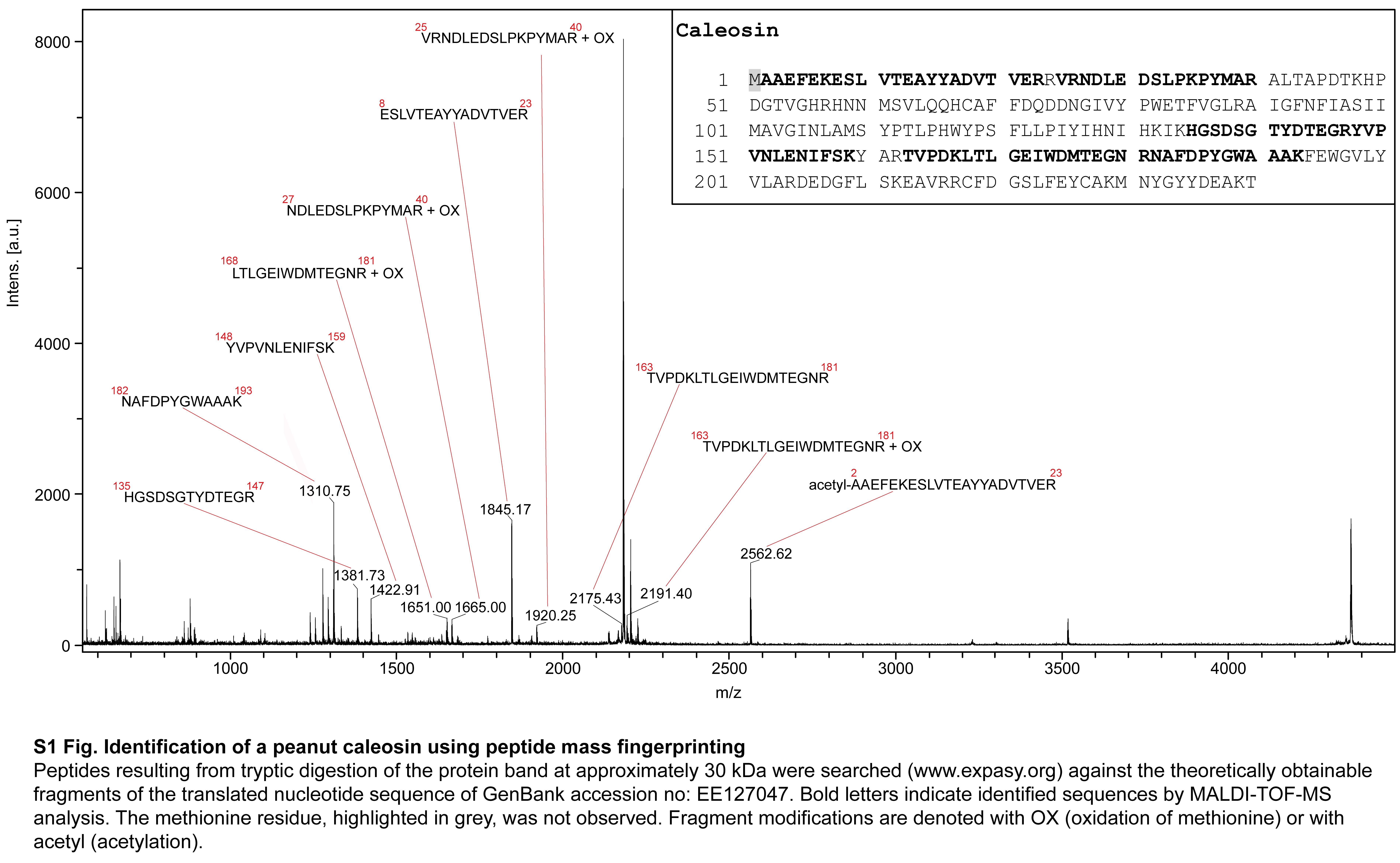

Supplement: S1 Fig — (TIF) [file pone.0123419.s001.tif]

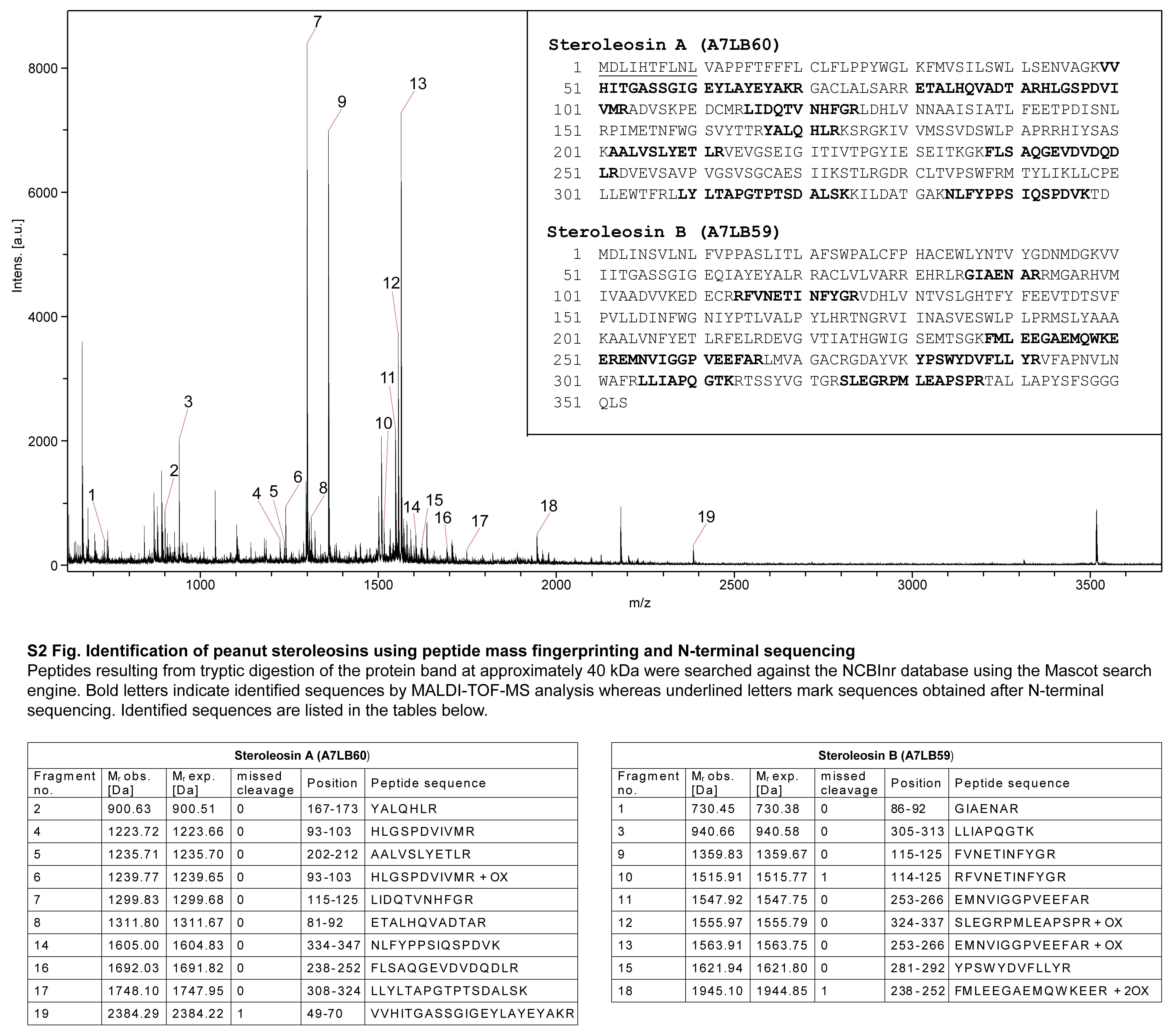

Supplement: S2 Fig — (TIF) [file pone.0123419.s002.tif]
